# Supplementary material for: A proposed de-identification framework for a cohort of children presenting at a health facility in Uganda
Source: PLOS Digit Health. 2022 Aug 24;1(8):e0000027. doi: 10.1371/journal.pdig.0000027 (PMC9931294; doi:10.1371/journal.pdig.0000027)
Supplement: S1 Appendix — (PDF) [file pdig.0000027.s001.pdf]

Functional definitions of key terms used in de-identification

Adversary	An individual attempting to re-identify one or more individuals in the dataset.
De-identification	A process of removing personal information from a dataset such that disclosure does not violate the privacy of individuals.
Direct identifier	Variable within a dataset that can uniquely identify an individual (e.g., first name, phone number, study ID).
Generalization	An approach to de-identification where attribute values of a variable are combined to create broader categories that will contain more records (e.g., participant ages get categorized into 12-month intervals).
Generalization hierarchies	A visual depiction of the regrouping of variables into broader categories used in generalization. At each consecutive hierarchy, categories should broaden.
k-anonymity model	A model to evaluate the risk of an individual being re-identified in a dataset. The model states that the risk of re-identifying a record in the dataset is equal to $\frac{1}{k}$, where k is the total number of records in the dataset that have identical quasi-identifier responses (with regard to the record in question).
Open Data	Data that is available for anyone to access, use, and share.
Re-identification risk threshold	An amount of risk of re-identifying individuals in the dataset that has been deemed acceptable. This is expressed as a probability or percentage (e.g., 0.05 or 5%).
Quasi identifier	Variables about research participants in the dataset than (1) an adversary is assumed to have background knowledge of and (2), can use, either individually, or in combination, to re-identify a record (e.g., sex, age, date of admission).
Suppression	An approach to de-identification where variables attribute values of a variable are deleted (e.g., the age of a specific participant is deleted and replaced with a blank cell).
