# Supplementary material for: A proposed de-identification framework for a cohort of children presenting at a health facility in Uganda
Source: PLOS Digit Health. 2022 Aug 24;1(8):e0000027. doi: 10.1371/journal.pdig.0000027 (PMC9931294; doi:10.1371/journal.pdig.0000027)
Supplement: Attachment — Submitted filename: Smart Triage De-Id Response to Reviewers 21JUNE22.docx [file pdig.0000027.s005.docx]

**Journal Requirements:

1. Please amend your detailed Financial Disclosure statement. This is published with the article. It must therefore be completed in full sentences and contain the exact wording you wish to be published. State the initials, alongside each funding source, of each author to receive each grant.**

I have included the following financial disclosure statement:

*“This research was supported by the Wellcome Trust Innovator Award (ID: 215695/B/19/Z), awarded to JMA and SA. The funders of the study had no role in study design, data collection, data analysis, data interpretation, or writing of the manuscript.”*

**2. Please update the completed 'Competing Interests' statement. Please declare all competing interests beginning with the statement “I have read the journal's policy and the authors of this manuscript have the following competing interests:”.**

I have included the following competing interests statement:

*“JMA serves as a section editor for PLOS Digital Health. The peer-review process was guided by an independent editor, and the authors have no other competing interests to declare.”*  **3. Please review your reference list to ensure that it is complete and correct. If you have cited papers that have been retracted, please include the rationale for doing so in the manuscript text, or remove these references and replace them with relevant current references. Any changes to the reference list should be mentioned in the rebuttal letter that accompanies your revised manuscript. If you need to cite a retracted article, indicate the article's retracted status in the References list and also include a citation and full reference for the retraction notice.**

The reference list is complete and correct.  **Additional Editor Comments (if provided):** N/A **[Note: HTML markup is below. Please do not edit.]

Reviewers' comments:

Reviewer's Responses to Questions

<font color="black"><b>Comments to the Author</b><br><br>
1. Does this manuscript meet PLOS Digital Health’s <a href="**[**https://journals.plos.org/digitalhealth/s/journal-information#loc-criteria-for-publication**](https://journals.plos.org/digitalhealth/s/journal-information#loc-criteria-for-publication)**" target="_blank">publication criteria</a>? Is the manuscript technically sound, and do the data support the conclusions? The manuscript must describe methodologically and ethically rigorous research with conclusions that are appropriately drawn based on the data presented.</font>


Reviewer #1: Yes

Reviewer #2: Yes

Reviewer #3: Yes

Reviewer #4: Yes

--------------------

<font color="black">2. Has the statistical analysis been performed appropriately and rigorously?</font>

Reviewer #1: Yes

Reviewer #2: I don't know

Reviewer #3: Yes

Reviewer #4: Yes

--------------------

<font color = "black">3. Have the authors made all data underlying the findings in their manuscript fully available (please refer to the Data Availability Statement at the start of the manuscript PDF file)?<br><br> The <a href="**[**https://journals.plos.org/digitalhealth/s/data-availability**](https://journals.plos.org/digitalhealth/s/data-availability)**" target="_blank">PLOS Data policy</a> requires authors to make all data underlying the findings described in their manuscript fully available without restriction, with rare exception. The data should be provided as part of the manuscript or its supporting information, or deposited to a public repository. For example, in addition to summary statistics, the data points behind means, medians and variance measures should be available. If there are restrictions on publicly sharing data—e.g. participant privacy or use of data from a third party—those must be specified.</font>

Reviewer #1: Yes

Reviewer #2: Yes

Reviewer #3: Yes

Reviewer #4: Yes

--------------------

<font color = "black">4. Is the manuscript presented in an intelligible fashion and written in standard English?<br/><br/>PLOS Digital Health does not copyedit accepted manuscripts, so the language in submitted articles must be clear, correct, and unambiguous. Any typographical or grammatical errors should be corrected at revision, so please note any specific errors here.</font>

Reviewer #1: Yes

Reviewer #2: Yes

Reviewer #3: Yes

Reviewer #4: Yes

--------------------

<font color = "black">5. Review Comments to the Author<br/><br/>Please use the space provided to explain your answers to the questions above. You may also include additional comments for the author, including concerns about dual publication, research ethics, or publication ethics. (Please upload your review as an attachment if it exceeds 20,000 characters)</font>

Reviewer #1: Thank you for conducting the study related to the protection of research participant data. Since privacy is extremely important nowadays, especially the research contained enormous personal information. The manuscript is well written and organized in detail. I have no further comment on the manuscript. Good luck to you.**

Thank you for taking the time to review our manuscript!

**Reviewer #2: This report includes a detailed description of a method used to protect identifiable information in research. The methods described in this study contribute new knowledge and could have public health implications by improving our knowledge on how to optimize data access whilst protecting identifiable health information, potentially fostering easier collaboration and hence data sharing. Further, by advocating for and outlining methods for implementing de-identification approaches, the findings have the ability to alleviate common concerns about data security and data protection to enhance public confidence in the scientific process. In summary, the report could alleviate barriers to conducting future research that will help to inform public health decisions.

However, some sections lacked clarity and require additional information for the reader to replicate the method. It is also unclear whether the approach described in the current study can be applied to many types of datasets or research questions, and how much it can affect the replicability of statistical analyses. Perhaps including a second example, as well as sharing the R code used to generate the de-identified dataset would greatly improve this paper.**

Thank you for your helpful review and feedback! I have addressed each of your comments in the table below.

**Reviewer 2 Comments**

**Summary**

**This report includes a detailed description of a method used to protect identifiable information in research. The methods described in this study contribute new knowledge and could have public health implications by improving our knowledge on how to optimize data access whilst protecting identifiable health information, potentially fostering easier collaboration and hence data sharing. Further, by advocating for and outlining methods for implementing de-identification approaches, the findings have the ability to alleviate common concerns about data security and data protection to enhance public confidence in the scientific process. In summary, the report could help to alleviate barriers to conducting future research that will help to inform public health decisions.**

**However, some sections lacked clarity and require additional information for the reader to replicate the method. It is also unclear whether the approach described in the current study can be applied to many types of datasets or research questions, and how much it can affect the replicability of statistical analyses. Perhaps including a second example, as well as sharing the R code used to generate the de-identified dataset would greatly improve this paper.**

Thank you for taking the time to review our manuscript! Below we have responded to each of your comments and suggestions.

**Strengths:
1.    Important topic with public health implications;**

**2.    Clearly defined methods and instructions for readers, with many tables to provide context;
3.    Recognition of the limitations of the approach.**

Thank you for this feedback.

**Specific comments/suggestions**

- **Abstract: No description of the dataset used for the analysis/demonstration of the method is provided in the abstract. At least the number of participants, some basic demographic info or a description of the variables used should be included here.**

Thank you for this suggestion. We have added the following sentence into the abstract:

*“We applied a standardized de-identification framework to a dataset comprised of 241 health related variables collected from a cohort of 1750 children with acute infections from Jinja Regional Referral Hospital in Eastern Uganda.”*

- **Line 141: “We have selected a semi-public release for the de-identified Smart Triage dataset to be published”. The authors did not give a rationale for selecting this type of data release, nor do they describe options for authors who do not have access to such access control platforms as the “Pediatric Sepsis Data CoLaboratory (Sepsis CoLab) Dataverse”. Are there other options for controlled data release available to researchers?**

Thank you for this feedback. There may be other options for controlled data release available to researchers, however, none that we are aware of or familiar with. We discussed the three traditional release models outlined by the Information and Privacy Commissioner of Ontario: public, semi-public, and non-public. We agree that it would be important for us to provide a rationale as to why we selected a semi-public release model and thus we have included the following paragraph in the manuscript:

*“Selection of a semi-public release model was based on best efforts to mitigate risk to participants while maximizing opportunities for data sharing and reuse. We decided that a public release model would not be appropriate given that the dataset was derived from a vulnerable pediatric cohort in a low-income country. On the other hand, a non-public release would considerably reduce data sharing opportunities. For these reasons, a semi-public release has been the general model adopted by the Pediatric Sepsis CoLab for sharing and distributing data.”*

- **Line 154: “usefulness in data analysis was considered” – The example provided is helpful to readers when making this judgement in their own analyses. However, a definition of ‘useful’ might be fitting here (for example: “A variable is defined as ‘useful’ where it does not have sufficient distinguishability but it is essential to the scientific objectives of the study”).**

Thank you. I have included an operational definition of “usefulness” in the manuscript:

*“Variables defined as useful are those that are essential to the scientific objectives of the study.”*

- **Paragraph starting on line 201: It is unclear whether this paragraph forms part of the ‘instructions’ described as Step 2 for the reader to use two independent investigators to classify the variables, or whether this represents part of an internal validation of the classification method described in the study. It currently reads as a method used for internal validation. Consider altering the language to make it clear that the method of de-identification will involve finding two researchers who must go through each variable (i.e. it should read as instructions to the reader).**

Thank you, we agree that this was unclear. For clarity, we have shifted this paragraph to directly follow the other paragraphs under “Step 2”. We have also changed the language to be more instructional:

*“The variable classification process is to be conducted independently by the two investigators most familiar with the data, and Cohen’s Kappa was computed to measure degree of agreement.”*

- **Lines 213-227: From table 2, I think some examples of variables with low, medium and high privacy thresholds would be helpful to provide context here.**

Thank you for this comment. Individual variables are not ranked as having low, medium, and high privacy thresholds. The privacy threshold ranking only applies to the dataset as a whole.

- **Line 247: Providing the R code used to conduct the de-identification process would greatly benefit the reader and improve the standardization of this process across studies. I understand that the package is references, however applying the code in the context of the named variables outlined in this study would greatly improve the reproducibility of this method.**

The data studied and the R code are available for download from the Pediatric Sepsis CoLab Dataverse. I have clarified this in the data availability statement:

*“The data studied and [the R] code are available on the Pediatric Sepsis CoLab Dataverse (https://doi.org/10.5683/SP3/MSTH98), subject to an application meeting the ethical and governance requirements of the CoLab (contact jessica.trawin@cw.bc.ca).”*

**General limitations**

1. **This method was developed and applied to only one dataset. The methods paper would be strengthened by applying the same approach to a different dataset with a different study objective.**

This is an excellent suggestion that should definitely be considered in future directions of this research. We decided not to include a second example in this current manuscript for the sake of maintaining simplicity and maximizing clarity to a subject that is quite complex for researchers/individuals who are not familiar with the subject.

2. **Similar to the above limitation, applying two different approaches of de-identification and comparing any changes to the reproducibility of the analysis would be a brilliant way to provide readers with further options.**

Again, we agree that this is an excellent suggestion for future directions. For this reason, we have made the data available for other researchers to look at using alternative approaches. We chose what was most simple for those new to the de-identification process, but we do acknowledge that there are many limitations and we look forward to other investigators trying new and improved methods for conducting data de-identification.

3. **There is limited information on handling dates. Is it possible to apply generalization or suppression methods to date variables? If not, this should be noted as a limitation.**

We agree that de-identification of “date” variable by applying generalization and suppression was not explored in this manuscript and thus should be acknowledged in the limitations. We have added the following to the manuscript:

*“Further, as all variables classified as dates in our study were converted into time intervals, we provided limited guidance on how to de-identify these types of variables using generalization and suppression.”*

The conversion of dates into time intervals however did add an additional layer of privacy protection as duration could start and end at any time in the study period. This is addressed in the discussion section of the manuscript:

*“While age is typically considered to be a quasi-identifier, in a study that has a duration of many months or years, converting the date of admission and date of birth provides generalization if these dates are suppressed and the date of admission could be anywhere within the study period. The same would be true of other time intervals such as length of stay.”*

**Reviewer #3: General comments
The manuscript is well written and all sub sections are clear to read.
The manuscript adequately describe the methods used.

Comment. Under limitations, Line 480 the sentence “Though it may appear concerning that many quasi-identifiers…” This sentence is not very clear. It is recommended to delete the word “concerning”.**

**Thank you**

Thank you for this comment. I have deleted the word “concerning” and the sentence now reads:

*“Though many quasi-identifiers were removed from the dataset, the quantity of collected data for these variables was not sufficient for meaningful analysis and the rarity in positive responses presented a high risk of privacy invasion (Table 3).”*

**Reviewer #4: Question 3 - While answer is Yes, the authors indicated that the data studied and code are available on the Pediatric Sepsis CoLab Dataverse and provided the link and contacts. Data is available subject to an application that meets the ethical and governance requirements.**

Yes, this is true. The application will take approximately five minutes of your time and it will take a few weeks to be approved by the governance committee. We are hopeful that we can move to a more rapid and secure process to provide access, but this will take time and funding. We have strongly advised our University to subscribe to the Vivli Platform that provides an end-to-end solution. **Additional comments:
This is a masterpiece paper, with the proposed methodology for data de-identification having a significant potential to positively contributing to research, even beyond the health sector. Any researcher who wants to comply with funder or publisher requests to make data open can easily follow the step-by-step guidelines proposed.**

The authors thank you for your review.  **--------------------

<font color = "black">6. PLOS authors have the option to publish the peer review history of their article (<a href="**[**https://journals.plos.org/digitalhealth/s/editorial-and-peer-review-process#loc-peer-review-history**](https://journals.plos.org/digitalhealth/s/editorial-and-peer-review-process#loc-peer-review-history)**" target="_blank">what does this mean?</a>). If published, this will include your full peer review and any attached files.<br><br>

<b>Do you want your identity to be public for this peer review?</b> If you choose “no”, your identity will remain anonymous but your review may still be made public.<br><br>

For information about this choice, including consent withdrawal, please see our <a href="**[**https://www.plos.org/privacy-policy**](https://www.plos.org/privacy-policy)**" target="_blank">Privacy Policy</a>.</font>


Reviewer #1: Yes: Yuen-ling LEUNG

Reviewer #2: No

Reviewer #3: No

Reviewer #4: Yes: Wellington Mushayi**
