# Supplementary material for: A proposed de-identification framework for a cohort of children presenting at a health facility in Uganda
Source: PLOS Digit Health. 2022 Aug 24;1(8):e0000027. doi: 10.1371/journal.pdig.0000027 (PMC9931294; doi:10.1371/journal.pdig.0000027)
Supplement: Attachment — Submitted filename: Reviewer Comments.docx [file pdig.0000027.s004.docx]

**Reviewer Comments**

**Summary**

This report includes a detailed description of a method used to protect identifiable information in research. The methods described in this study contribute new knowledge and could have public health implications by improving our knowledge on how to optimize data access whilst protecting identifiable health information, potentially fostering easier collaboration and hence data sharing. Further, by advocating for and outlining methods for implementing de-identification approaches, the findings have the ability to alleviate common concerns about data security and data protection to enhance public confidence in the scientific process. In summary, the report could help to alleviate barriers to conducting future research that will help to inform public health decisions.

However, some sections lacked clarity and require additional information for the reader to replicate the method. It is also unclear whether the approach described in the current study can be applied to many types of datasets or research questions, and how much it can affect the replicability of statistical analyses. Perhaps including a second example, as well as sharing the R code used to generate the de-identified dataset would greatly improve this paper.

**Strengths:**
1.    Important topic with public health implications;

2.    Clearly defined methods and instructions for readers, with many tables to provide context;
3.    Recognition of the limitations of the approach.

**Specific comments/suggestions**

- Abstract: No description of the dataset used for the analysis/demonstration of the method is provided in the abstract. At least the number of participants, some basic demographic info or a description of the variables used should be included here.
- Line 141: “We have selected a semi-public release for the de-identified Smart Triage dataset to be published”. The authors did not give a rationale for selecting this type of data release, nor do they describe options for authors who do not have access to such access control platforms as the “Pediatric Sepsis Data CoLaboratory (Sepsis CoLab) Dataverse”. Are there other options for controlled data release available to researchers?
- Line 154: “usefulness in data analysis was considered” – The example provided is helpful to readers when making this judgement in their own analyses. However, a definition of ‘useful’ might be fitting here (for example: “A variable is defined as ‘useful’ where it does not have sufficient distinguishability but it is essential to the scientific objectives of the study”).
- Paragraph starting on line 201: It is unclear whether this paragraph forms part of the ‘instructions’ described as Step 2 for the reader to use two independent investigators to classify the variables, or whether this represents part of an internal validation of the classification method described in the study. It currently reads as a method used for internal validation. Consider altering the language to make it clear that the method of de-identification will involve finding two researchers who must go through each variable (i.e. it should read as instructions to the reader).
- Lines 213-227: From table 2, I think some examples of variables with low, medium and high privacy thresholds would be helpful to provide context here.
- Line 247: Providing the R code used to conduct the de-identification process would greatly benefit the reader and improve the standardization of this process across studies. I understand that the package is references, however applying the code in the context of the named variables outlined in this study would greatly improve the reproducibility of this method.

**General limitations**

1. This method was developed and applied to only one dataset. The methods paper would be strengthened by applying the same approach to a different dataset with a different study objective.
2. Similar to the above limitation, applying two different approaches of de-identification and comparing any changes to the reproducibility of the analysis would be a brilliant way to provide readers with further options.
3. There is limited information on handling dates. Is it possible to apply generalization or suppression methods to date variables? If not, this should be noted as a limitation.
